# Applications of Molecular Diagnostic Testing in Food Allergy

**DOI:** 10.1007/s11882-015-0557-6

**Published:** 2015-08-02

**Authors:** Karin Hoffmann-Sommergruber, Sabine Pfeifer, Merima Bublin

**Affiliations:** Department of Pathophysiology and Allergy Research, Medical University of Vienna, AKH-EBO3Q, Waehringer Guertel 18-20, 1090 Vienna, Austria

**Keywords:** Allergens, Allergy diagnosis, Component-resolved diagnosis, Food allergy, IgE antibodies, Molecular diagnosis

## Abstract

IgE-mediated food allergy is a relevant health problem inducing symptoms ranging from mild local reactions up to severe life-threatening situations. Currently, no immunotherapy is available and avoidance of the incriminating food is the method of choice. Therefore, reliable diagnostic tools to formulate dietary recommendations and to avoid unnecessary exclusion diets for the individual patient are urgently needed. This review provides an update on the current knowledge on food allergens and their application in various diagnostic approaches such as skin prick test, basophil activation test, and serum IgE testing. Furthermore, these new approaches are discussed and compared to conventional extract-based assays and correlated to the gold standard of food allergy diagnosis, the double-blind placebo-controlled food challenge. Finally, the application of food allergens for preventive measurements such as allergen detection assays and the determination of threshold levels for allergen levels are discussed.

## Introduction

Allergic diseases are regarded as a relevant global burden to society and healthcare systems. The prevalence for asthma, allergic rhinitis, and atopic eczema has indeed increased in the recent past. However, for IgE-mediated food allergy, the picture is less clear. A recent systematic review and meta-analysis of food allergy in Europe identified a 17.3 % pooled lifetime prevalence of self-reported food allergy and a point prevalence of 5.9 % [1, 2]. In contrast, the point prevalence of food allergy (FA) diagnosis based on food challenges was around 0.9 %, yet differing on the food allergen source and depending on the age. While for diagnosis of food allergy, double-blind placebo-controlled food challenge (DBPCFC) is regarded as the gold standard; a careful case history is equally mandatory. The detection of allergen-specific IgE antibodies completes the state-of-the-art diagnosis of food allergy [3]. Besides symptomatic treatment, there is no causative immunotherapy available. Currently, avoiding the causative allergen sources is the method of choice.

In food allergy, two different routes of sensitization are known. While in primary food allergy, the atopic individual becomes sensitized against the culprit dietary protein through the gastrointestinal tract, and in secondary food allergy, sensitization occurs via pollen or latex allergens and the allergic reaction is induced by cross-reactive homologous food allergens [4].

The diagnostic relevance of specific IgE testing strongly depends on the quality of the analyte used. For more than 100 years, total extracts obtained from allergen sources were used. It is well known that these extracts are not very well standardized and may lack certain allergens, e.g., due to enzymatic activity [3].

During the past 25 years, a plethora of individual allergens has been identified and their physicochemical properties analyzed. Especially, up-to-date molecular biology methods facilitated the production of recombinant allergens for in vitro diagnosis. The determination of specific IgE to different allergens is called molecular diagnosis or component-resolved diagnosis (CRD).

Molecular diagnosis will help to increase the sensitivity of the testing and, on the other hand, provides a more-detailed patient-tailored risk profile. This in turn can help to formulate improved dietary recommendations and reduce unnecessary exclusion diets [3].

However, it has to be stated that most allergens described in CRD studies so far have been applied in experimental settings. Therefore, the recent EAACI Guidelines on the Diagnosis and Management of Food Allergy clearly emphasized the urgent need for well-designed randomized controlled studies to assess the diagnostic value of CRD-based tests [3].

## Allergen Extract Versus Single Allergens

### Production and Purification of Allergens for Molecular Diagnosis

Allergens used for CRD can be obtained from either natural sources or expressed in heterologous expression systems. The decision whether to purify natural or recombinant allergens has to be made on a case-by-case approach and depends on a number of aspects. For example, the presence and impact of any post translational modification on the IgE binding activity of the target protein need to be evaluated throughout the purification process. If a given allergen consists of a number of isoforms all relevant for allergy diagnosis, then the natural mix of proteins is to be preferred. In contrast, if one isoform is representative for IgE-based diagnosis, then the recombinant production can be chosen. Similarly, if the natural protein is sensitive to enzymatic degradation during the extraction process, then heterologous expression systems can be advantageous. If the correct three-dimensional structure of the protein is crucial for the known interaction with IgE antibodies, then either suitable expression systems or purification from natural sources can be chosen [5, 6]. Equally important is the development of a suitable purification protocol of the allergen of interest. Finally, the protein has to be tested for its physicochemical properties such as stability, purity, correct primary, secondary, and tertiary structure, enzymatic activity, and IgE binding capacity [7, 6]. As a proof of concept, an allergen library was developed within the EU-funded project EuroPrevall, collecting highly pure and well-characterized food allergens from the most important food allergen sources. Up to date, the integrity of the purified allergen batches was assessed by technologies such as HPLC, mass spectrometry, and infrared spectroscopy. CD spectroscopy and 1D-NMR can be applied to analyze the secondary structure of allergens [8].

As stated above, a considerable number of allergens have been identified from animal- and plant-derived foods. Consecutively, a number of allergen databases were established to collect and update the existing data on allergens, their physicochemical features, and allergenic relevance. The IUIS allergen database (http://www.allergen.org) is the official source, providing a systematic nomenclature for allergens, taking care of identification of allergens, and upon positive evaluation of the allergen nomenclature subcommittee, the official allergen designation is granted [9, 10]. The Allergome database (http://www.allergome.org) is a continuously updated non-peer-reviewed literature collection about all aspects of allergens, while the AllergenOnline (http://www.allergenonline.org) provides peer-reviewed information about allergens and sequence comparison tools for allergenic risk assessment. SDAP, the structural database of allergens, collects all the known structures of allergens (http://fermi.utmb.edu/SDAP/index.html), while others such as the InformAll link clinical data with allergens (http://www.inflammation-repair.manchester.ac.uk/informAll/).

Analyzing all the existing allergen sequences, it became evident that only a minority of all known protein families contain intrinsic properties that render them allergenic [11–13].

## Tests for Food Allergy Diagnosis

In the following, the application of allergen-specific testing for food allergy diagnosis will be discussed. Furthermore, an overview on the most important plant-derived food allergen sources and their respective allergen panels will be presented.

### Skin Prick Testing

In vivo skin prick testing for food allergy diagnosis is routinely performed. The food extracts used for skin prick testing (SPT) are not all standardized and may vary from batch to batch missing some allergens [14–16]. Therefore, prick-to-prick testing with the food source is applied in daily practice, especially when testing plant food allergen sources (fresh fruits, vegetables, nuts).

In proof-of-concept studies, purified recombinant fruit allergens from apple and peach were tested in SPTs. Bolhaar and colleagues applied purified recombinant apple allergen Mal d 1, the Bet v 1 homologue, by SPT in 14 patients using serial dilutions ranging from 0.02, 0.2, 2, 20, to 100 μg/mL. Positive skin reactions were observed in a dose-dependent manner starting with the lowest concentration of 2 μg/mL [17]. In a similar study, purified recombinant Mal d 4, the apple profilin, was tested in 5 patients in the same serial dilutions 0.02, 0.2, 2, 20, and 100 μg/mL, giving positive reactions at a the dose of 0.2 μg/mL [18].

Garcia et al. performed SPTs with four peach extracts containing 0.4, 2, 10, and 50 μg/mL of Pru p 3, the non-specific lipid transfer protein (nsLTP) from peach [19], during a sublingual immunotherapy trial with natural Pru p 3. They showed that after 6 months of SLIT, the active group presented a significant decrease (5.3 times) in SPTs, which correlated with a significant increased tolerance of peach intake [19].

In a recent study, Kollmann et al. used purified recombinant Mal d 1 and Bet v 1 (birch pollen allergen) and birch pollen extract (BPE) in SPTs [20•]. For this study, the recombinant allergen was produced under good manufacturing practice (GMP) conditions suitable for clinical trials. Twenty out of 21 patients had a positive reaction to rMal d 1 (50 μg/mL) and to fresh apple. The overall sensitivity of the SPT was 95 % for rMal d 1 [20•].

There are not yet purified recombinant allergens approved for SPTs in clinical routine, while for some European countries, purified natural peach nsLTP (Pru p 3) and purified natural date profilin can be used [21].

Peeters and colleagues applied purified natural peanut allergens, Ara h 1, Ara h 2, Ara h 3, and Ara h 6, in SPTs in a collective of 30 peanut-allergic patients in serial dilutions ranging from 100 μg/mL down to 0.01 μg/mL [22]. The majority of the patients with severe reactions to peanuts had positive SPTs to Ara h 2 and Ara h 6, even at low concentrations. However, SPT reactions to Ara h 1 and Ara h 3, at higher concentrations, were also indicative for severe reactions [22]. In a pediatric study, the same set of natural peanut allergens was used for SPTs and provided a similar reactivity pattern as in the adult study group, with Ara h 2 and Ara h 6 inducing in most of the patients a positive reaction, while this was less pronounced for Ara h 1 and Ara h 3, respectively [23]. However, positive allergen-specific SPTs could not be related to the severity of peanut allergy.

### Serum IgE Testing

Extract-based in vitro testing for specific IgE antibodies has been used for a long time and is still in use for routine diagnosis. In the past three decades, advanced molecular biology methods enabled a dramatic increase in our knowledge on individual allergens and their potential application in diagnosis and therapy. The application of individual purified allergens for in vitro diagnosis has been designated molecular diagnosis or CRD.

One application of purified, well-characterized allergens is their use as a certified reference material for existing and approved in vitro diagnostics. The European Union (EU)-funded project CREATE used well-defined inhalant allergens and provided a proof-of-concept study to improve standardization of existing extract-based diagnostics [24]. However, while this has been shown for inhalant allergies, no such tools are available for food allergy to date. One option is to spike a given food extract with one or several individual allergens. So far, only one example has entered the market, namely Cor a 1.04, the Bet v 1 homologous food allergen from hazelnut, which was added to hazelnut extract for improved in vitro diagnosis [25]. While the Cor a 1.04-specific IgE antibody detection improved upon spiking, no assay interference due to the spiking event was observed for the other allergens present in the hazelnut extract. In a clinical study, the results obtained with spiked hazelnut extract for specific IgE antibodies were compared with DBPCFCs in hazelnut-allergic children [26]. While the negative predictive value of in vitro diagnosis was improved with the spiked hazelnut extract, the positive predictive value was rather moderate to decreased [26].

Recently, CRD for hazelnut and peanut allergy was compared to the outcome of DBPCFCs. For peanut allergy, Eller and Bindslev-Jensen identified anti-Ara h 2 IgE >1.63 kU/L as a clear decision point with both optimal specificity and high sensitivity to predict clinically relevant peanut allergy [27••]. In addition, applying this cutoff value, the number of necessary DBPCFCs could be significantly reduced. In contrast, Beyer and co-authors identified 14.4 kU/L anti-Ara h 2 IgE to be 90 % predictive for a positive peanut challenge [28••]. For hazelnut positive challenge outcome, these authors calculated Cor a 14-specific IgE at 47.8 kU/L to be 90 % predictive [28••].

### Basophil Activation Test

Cellular tests are available to verify the presence of allergen-specific IgE in patients’ sera and the ability to activate basophils. These tests either use autologous cells from the donor or established cell lines. After passive sensitization with serum and the addition of allergen extracts or allergens, cell activation can be measured by the determination of histamine or sulfidoleukotriene release. Alternatively, expression of a surface marker such as CD63 or CD203c is detected by fluorescence-activated cell sorting (FACS) analysis. CD63, a glycoprotein, is present only in low levels in resting basophils. However, upon activation, it is upregulated. Similarly, CD203c, an ecto-enzyme, is expressed on the cell membrane of human basophils and mast cells and becomes upregulated upon cross-linking of the Fc-epsilon receptor alpha. Erdmann and co-authors tested sera from 32 patients with Bet v 1-related plant food allergies. The recombinant allergens Api g 1 from celery, Dau c 1 from carrot, and Mal d 1 from apple were applied in the basophil activation test (BAT) assay, and CD63 upregulation was determined by FACS analysis. In this study, sensitivity for BAT ranged from 65 to 75 % and specificity from 68 to 100 % [29].

Sato and co-workers evaluated the CD203c expression on basophils from 71 egg- and milk-allergic children in the BAT assay [30]. In parallel, egg extract and Gal d 1, ovomucoid, and cow’s milk extract and Bos d 8, casein, were applied. The authors determined the CD203c stimulation index and the threshold of CD203c expression. In their study population, they found no difference in sensitivity, specificity, and positive predictive values (PPV), when comparing milk extract to Bos d 8. In hen’s egg allergy, sensitivity, specificity, and PPV for Gal d 1 were superior as compared to those in hen’s egg extract [30].

Similar studies were also performed in peanut-allergic children. Glaumann and co-authors evaluated the basophil allergen threshold sensitivity, called CD-sens, and peanut allergen-specific IgE antibody levels in relation to DBPCFC outcome [31•]. Basophil tests were performed with both peanut extract and purified rAra h 2 and yielded positive results in 92 % of the tested samples and were in agreement with a positive DBPCFC. Negative outcome in CD-sens was paralleled by negative results in DBPCFCs. Finally, specific IgE raised against Ara h 1, Ara h 2, and Ara h 3 was clearly associated with peanut allergy [31•].

Santos and co-workers compared the threshold of allergic reactions to peanuts with peanut extract basophil reactivity and identified clear associations [32].

For milk allergy, CRD was performed in milk-allergic patients and compared to the outcome of the basophil degranulation tests [33]. Despite specific IgE directed against milk allergens, the basophil degranulation test could discriminate between milk-allergic patients and patients still sensitized but that had already outgrown. Ford and co-workers performed another study, including milk-allergic children and assessed, whether they had already acquired tolerance by specific IgE testing and BAT assays using baked milk antigens. Again, the results from the BAT test proved useful in the identification of different phenotypes of the patients [34].

## Food Allergen Panels for CRD

For some foods, a range of allergens are available for CRD, while for others, only single allergens have been identified to date. In the recent past, efforts were undertaken to perform CRD studies in well-characterized patients with positive DBPCFCs. These studies contribute to compile a patient-tailored risk profile and allow to relate distinct sensitization patterns with either mild or severe and/or generalized symptoms. They also allow to identify cross-sensitizations such as pollen allergens and help to discriminate between primary and secondary food allergy. In addition, these studies provide useful information about the sensitivity and specificity of these allergen-specific IgE testing. In the following, the selected examples of plant food allergen panels which are summarized in Table 1 will be briefly discussed.

**Table 1:** Selected studies on allergen panels used for CRD in plant food allergies

Source	Allergens	Study group	Reference
Fruits			
Apple *(Malus domestica)*	Mal d 1, Mal d 2, Mal d 3, Mal d 4	389 apple-allergic patients (AT, NL, ES, IT)	Fernandez-Rivas et al. 2003 [36]
Peach *(Prunus persica)*	Pru p 1, Pru p 3, Pru p 4	76 peach-allergic adult patients (ES)	Fernandez-Rivas et al. 2003 [37];
		57 peach-allergic pediatric patients (ES)	Boyano-Martinez et al. 2013 [38••]
		148 peach-allergic adolescent/adult patients (IT)	Pastorello et al. 2013 [39]
Kiwifruit *(Actinidia deliciosa)*	Act d 1, Act d 2, Act d 4, Act d 5, Act d 6, Act d 7, Act d 8, Act d 9, Act d 11	92 kiwifruit-allergic patients	Palacin et al. 2008 [59]
		30 kiwifruit-allergic patients (CH)	Bublin et al. 2013 [41]
		311 kiwifruit-allergic patients (EU-wide study)	Le et al. 2013 [42••]^a^
Vegetables			
Carrot *(Daucus carota)*	Dau c 1, Dau c 4, Dau c 5	49 carrot-allergic patients (DK, CH, ES)	Ballmer-Weber et al. 2012 [46••]
Celery (*Apium graveolens)*	Api g 1, Api g 4, Api g 5	24 celeriac-allergic patients (CH)	Bauermeister et al. 2009 [43]
Tree nuts			
Hazelnut *(Corylus avellana)*	Cor a 1, Cor a 2, Cor a 8, Cor a 9, Cor a 11, Cor a 12, Cor a 14	52 hazelnut-allergic patients (DK, CH, ES)	Hansen et al. 2009 [47]
		161 hazelnut-allergic patients (NL)	Masthoff et al. 2013 [48••]
		423 patients (EU-wide study)	Datema et al. 2015 [49••]
Fabales			
Peanut *(Arachis hypogaea)*	Ara h 1, Ara h 2, Ara h 3, Ara h 6, Ara h 8, Ara h 9	Population-based birth cohort study; 933 children; (UK) 150 peanut-allergic patients (adults; EU-wide study)	Nicolaou et al. 2010 [51••] Ballmer-Weber et al. 2015 [57•]

The selected studies performed at least in a subset of patients DBPCFCs or open challenges for diagnosis of food allergy

^a^Outpatient clinic study: no challenges were performed

### Apple and Peach

Recent prevalence data for fruit allergies identified peach and apple as the most frequent sensitizers in Europe [35]. Four allergens have been officially designated for apple. These proteins were applied for CRD studies in the SAFE study. The Bet v 1 homologue, Mal d 1, was the major allergen in Central Europe (Netherlands, Austria) and linked with Fagales pollen allergy. In contrast, sensitization to the nsLTP, Mal d 3, was frequent in Southern Europe (Spain, Italy) and linked with severe allergic symptoms [36]. Sensitization to profilin, Mal d 4, is evenly distributed across Europe, associated mostly with grass and tree pollen allergy. For the thaumatin-like protein, Mal d 2, the dataset is less clear.

Peach allergy is dominant in Southern Europe and correlates with high consumption rates of fresh fruits throughout the year. Pru p 3, the nsLTP, is the major allergen in clinically relevant peach allergy [37] and accumulates in the peach peel. Boyano-Martinez assessed a high Pru p 3 sensitization rate in children and confirmed that the majority of the patients tolerated peach pulp [38••]. Sensitization to Pru p 3 starts earlier in life as compared to Pru p 1 and Pru p 4, the Bet v 1 homologue and the profilin, respectively [39].

### Kiwifruit

The number of kiwifruit-allergic patients has remarkably increased in the last two decades, and the actual prevalence rate of sensitization in Europe is 5.2 % [35]. Since the extract-based IgE tests were of low sensitivity, efforts were undertaken to apply the existing kiwifruit allergen panel for CRD. Bublin and co-workers tested 237 sera from kiwifruit-allergic patients in the microarray format. The panel of allergens showed a diagnostic sensitivity of 66 %, a specificity of 56 %, and a positive predictive value of 73 % [40]. Actinidin, Act d 1, from kiwifruit is regarded as a marker for kiwifruit monosensitization and linked with generalized symptoms. In contrast, pollen-related kiwifruit allergy correlates with sensitization to the Bet v 1 homologue and profilin, Act d 8 and Act d 9, respectively [41]. Testing the large panel of different kiwifruit allergens showed that some allergens sensitize only in rare cases and may be specific for certain geographic area, such as Act d 6 [40]. In a European study, 311 sera from kiwifruit-allergic patients were tested. Comparing different geographic regions, sensitization to Act d 9, the kiwifruit nsLTP, was predominant for Southern Europe [42••]. Act d 1 was identified as a marker for severe symptoms upon kiwifruit consumption.

### Vegetables

Celery is a frequent cause for food allergic reactions, especially in Europe with a sensitization prevalence of 6.3 % [35]. Food allergic symptoms can range from mild local reactions, usually restricted to the oral mucosa, up to severe anaphylactic reactions. Sensitization to the pollen-related allergens Api g 1, the birch pollen homologue, and Api g 4, the profilin, are usually found in patients with concomitant tree pollen allergy and account for rather mild symptoms. Api g 5 is a flavin adenine dinucleotide (FAD) containing oxidase and contains N-glycans, which account for the majority of its IgE binding capacity [43]. Api g 2 and Api g 6 are both nsLTPs, differently expressed throughout the plant tissue [44, 45]. So far, little is known about their relevance as major or minor allergens. However, it seems that the celeriac allergen panel is still incomplete; a marker allergen responsible for the mugwort–celery cross-reactivity, usually known to be linked with severe symptoms in allergic patients, is especially missing.

In parallel, carrot allergy is predominant in areas where Fagales pollen exposure is abundant. This cross-reactivity is due to the Bet v 1 homologue, Dau c 1, and the profilin, Dau c 4 [46••]. The diagnostic relevance of additional carrot allergens, such as Dau c 5, the isoflavone reductase, is not yet determined.

### Tree Nuts

Among tree nuts, hazelnuts are a common source of food allergies, with and without concomitant pollen allergy with an overall sensitization prevalence of 9.3 % in Europe [35]. Hansen and co-authors investigated the sensitization profiles from 52 hazelnut-allergic patients from Denmark, Switzerland, and Spain [47]. While the Bet v 1 homologue, Cor a 1.04, was predominant in Northern and Central Europe, the sensitization to Cor a 8, the nsLTP from hazelnut, dominated in Spain. Profilin, Cor a 2, sensitization was evenly distributed in all countries (40–45 %). Only in 2 % sensitization to Cor a 11 was detected. Masthoff et al. investigated a total of 161 hazelnut-allergic patients, including children and adults, and identified sensitization to Cor a 14, the 2S albumin, and Cor a 9, the 11S globulin, as a marker for rather severe allergy symptoms [48••]. In a recent European study, Datema et al. used 7 hazelnut allergens to test sera from 423 hazelnut-allergic patients [49••]. IgE levels against Cor a 1 were the highest ones as compared to other hazelnut allergens. Cor a 8 sensitization was dominant in Greece and Spain. Cor a 14 sensitization was highly correlated with sensitization to Cor a 9. Cor a 11 sensitization was generally low, in all study centers. However, sensitization to Cor a 12, the oleosin from hazelnut, was detected in 10–25 % of the patients in most of the centers. Interestingly, sensitization to seed storage proteins and oleosins correlated with sensitization to other nuts, seeds, and pollens [49••].

### Peanuts

Peanut is known as a highly allergenic food, tentatively inducing severe, even life-threatening symptoms. In some patients, even minute amounts of peanuts can induce allergic reactions. Therefore, improved in vitro diagnosis could help to reduce the number of DBPCFCs and help to determine individual threshold levels for the high-risk patients. Burney and co-authors identified a sensitization prevalence of 2.7 % [35], yet the prevalence of clinical relevant peanut allergy is expected to be around 1 % [50]. In order to get a deeper insight into actual population-based data on peanut allergy, Nicolaou and co-workers investigated the prevalence of peanut allergy versus tolerance in children at the age of 8 years (total 933 participants) [51••]. The sensitization profiles were assessed using peanut allergens, Ara h 1, Ara h 2, Ara h 3, and Ara h 8, and SPTs were performed with peanut extract. Within this collective, 11.8 % had specific IgE to at least one of the peanut allergens. However, only 22.4 % out of the sensitized individuals had a clinically relevant peanut allergy, as assessed by DBPCFCs. When correlating the sensitization profiles with the DBPCFC results, Ara h 2, the 2S albumin from peanut, was the best predictor for clinical peanut allergy. Since then, a number of studies confirmed the relevance of Ara h 2 as a predictor for peanut allergy in both pediatric and adult study groups [52, 53•, 27••, 28••]. When Ara h 6, another 2S albumin from peanut, became available for molecular diagnosis, it was found to be an equally important predictor for peanut allergy [54•, 55]. Specific IgE directed against Ara h 1, the 7S globulin, and Ara h 3, 11S globulin from peanut, are additional relevant markers for peanut allergy [56, 57•]. A recent European study analyzed the sensitization patterns of peanut allergy patients and confirmed the predictive value of spIgE against Ara h 2. Sensitization to Ara h 8 (Bet v 1 homologue) and Ara h 9 (nsLTP) was observed in different geographical regions and was more frequently found in peanut-tolerant subjects. Sensitization to Ara h 1 and Ara h 2 was exclusively observed in early onset of peanut allergy [57•].

## Purified Food Allergens for Food Safety Aspects

In order to protect the allergic consumer from unintended exposure to allergenic food, legislation on allergen labeling has been set in place in many countries across the world. Consecutively, food industry has started initiatives to disseminate best practices for allergenic risk assessment and management among food producers. However, to implement risk assessment strategies during food production, processing, and packaging, reliable allergen detection assays and certified reference materials are still lacking. In addition, an agreed definition of a tolerable allergenic risk including threshold levels for each allergenic food is not yet in place [58]. For all these preventive measurements, purified well-defined food allergens can be used, and by replacing total extracts or spiking, total extracts with individual allergen test systems could be improved and provide more accurate determinations.

## Conclusions

In the last two decades, our knowledge on individual allergens has dramatically increased. As a consequence, the application of well-defined molecules for in vitro diagnosis was started for the most important food allergen sources. While for some foods, the allergen panels are considerable complex, and for others, only a few allergens are identified yet. Nevertheless, it became evident that molecular diagnosis provides additional information as compared to conventional extract-based testing. It enables to identify cross-sensitization to inhalant allergen sources such as pollens. Furthermore, for some allergens, it emerged that they predominantly evoke mild allergic symptoms, while for others, they are inducers for severe symptoms. Nevertheless, these molecular diagnosis approach needs to be assessed on a food-by-food approach and the sensitivity and specificity of this test has to be compared to the conventional diagnostic tests, including the DBPCFCs. Therefore, more clinical studies are needed for assessing the relevance of molecular diagnosis for each food allergen source.
